# Negative Ion Formation by the Thermal Surface Ionization of Oxygen-Bearing Gases (O_2_, CO_2_, CO, NO, and NO_2_)

**DOI:** 10.3390/molecules30112420

**Published:** 2025-05-31

**Authors:** Patryk Gontarz, Andrzej Pelc

**Affiliations:** Mass Spectrometry Laboratory, Department of Biophysics, Institute of Physics, Maria Curie-Skłodowska University, Pl. M. Curie-Skłodowskiej 1, 20-031 Lublin, Poland; patryk.gontarz@mail.umcs.pl

**Keywords:** electron attachment, dissociation, negative ion, thermal surface ionization, mass spectrometry

## Abstract

The formation of the oxygen negative ion O^−^ from simple molecules such as O_2_, CO_2_, CO, NO, and NO_2_ is of fundamental importance in environmental, atmospheric, and biological processes. This study investigates the mechanisms of O^−^ ion generation from these gases by analyzing the dependence of O^−^ ion current intensity on filament temperature. Optimum temperatures for O^−^ formation were identified for each gas, ranging from 1548 to 1721 °C. A comparison with the calculated thermal decomposition temperatures of the respective compounds indicates that distinct ion formation pathways are involved. For NO_2_, the process likely involves a two-step dissociation mechanism, with molecular oxygen (O_2_) formed in the first step, subsequently dissociating into O^−^ and O atoms. In contrast, for CO, O^−^ formation predominantly occurs through electron capture followed by molecular dissociation. These findings underscore the complex nature of negative surface ionization, which includes contributions from the capture of emitted from the cathode electrons by molecules.

## 1. Introduction

Simple oxygen-containing molecules, such as oxygen (O_2_), carbon dioxide (CO_2_), carbon monoxide (CO), nitric oxide (NO), and nitrogen dioxide (NO_2_), play pivotal roles in various chemical, environmental, and biological processes. These small, diatomic, or triatomic molecules are fundamental components of the Earth’s atmosphere, involved in critical phenomena ranging from greenhouse gas effects and air pollution to biochemical signaling and industrial processes [1,2]. These molecules, as well as their ions, have also been detected in space, for example, in the atmosphere of planets [3,4]. Oxygen itself, as an element which is a component of all the molecules studied here, is the third most common element in the universe (after hydrogen and helium). Similarly, carbon and nitrogen are high on the list of abundance of elements, occupying 4th and 7th place, respectively.

Oxygen (O_2_) is a molecule essential for most life forms on Earth, playing an important role in various biological and chemical processes. Oxygen is a crucial reactant in aerobic respiration [5] and is produced as a byproduct of photosynthesis. Moreover, oxygen is also essential for the formation of reactive oxygen species, which play a dual role in biological systems. While excessive oxygen species can cause oxidative stress and damage cellular components, at controlled levels, they serve as signaling molecules involved in processes like immune response and apoptosis [5,6]. In the upper atmosphere, oxygen can react to form ozone (O_3_), which plays a critical role in protecting living organisms from harmful ultraviolet (UV) radiation.

Carbon dioxide (CO_2_) is a well-known greenhouse gas, primarily produced by the combustion of fossil fuels and biological respiration [7]. Its increasing concentration in the atmosphere has been a major driver of global climate change, leading to significant interest in understanding its sources, sinks, and mechanisms for mitigation. CO_2_ also plays a key role in photosynthesis, serving as a carbon source for plants and thus influencing the global carbon cycle.

Carbon monoxide (CO) is produced both naturally and anthropogenically, notably from incomplete combustion of carbon-containing fuels [8]. While considered a pollutant, CO is also a precursor to tropospheric ozone (similarly to NO and NO_2_) and plays a complex role in atmospheric chemistry by interacting with hydroxyl radicals (OH), thus affecting the lifetimes of other atmospheric constituents [9].

Nitric oxide (NO) and nitrogen dioxide (NO_2_) are collectively referred to nitrogen oxides (NO_x_) and are primarily emitted from combustion processes. NO is a free radical and a critical intermediary in the atmospheric formation of both smog and acid rain. It also plays a crucial role in biological signaling, particularly in neurotransmission [2]. NO_2_, a product of NO oxidation, is a key atmospheric oxidant and a significant component of urban air pollution, contributing to respiratory problems and environmental degradation through the formation of secondary pollutants like ozone and particulate matter [10].

Understanding of properties, behavior, and interactions of these molecules is essential across multiple disciplines, including environmental science, atmospheric chemistry, medicine, and materials science. A deeper understanding of these molecules’ behavior also necessitates investigating their interaction with electron and associated with processes, like ion formation or electron attachment. The interactions of O_2_, CO_2_, CO, NO, and NO_2_, molecules with electrons significantly influence the chemistry and reactivity of these gases especially in atmospheric and plasma environments. Electron attachment, in particular, is a process where a free electron is captured by a molecule, resulting (at first step) in the formation of the molecular negative ion, a so-called transient negative anion (TNI), in most cases in the excited state. Then, this anion may dissociate to negatively charged and neutral fragments (molecules or atoms) in dissociative electron attachment (DEA) process. Another possibility for TNI deexcitation is to detach an extra electron in the autodetachment process [11]. Investigations of electron driven processes are crucial for understanding molecular stability, reaction dynamics, and the formation of reactive species. The processes involving the attachment of electrons to the gas molecules under consideration have been extensively studied [12,13,14,15,16,17,18,19,20,21,22,23,24,25,26,27,28,29,30]. It should be noted that in previous investigations on the formation of negative ions from O_2_, CO_2_, CO, NO, and NO_2_, anions were primarily generated through the interaction of molecular beams with electrons of defined energies. The resulting ions were subsequently identified using a mass spectrometer. Most recent studies indicate that O^−^ is the only anion formed via electron capture in the gases studied. Further details regarding the formation of the O^−^ anion from these gases (such as resonance energies) are provided in the beginning of the section discussing the experimental results. Here, we describe results of our studies of the formation of negative anions from simple oxygen-bearing molecules by means of its ionization on hot metal surface—negative thermal ionization. It is also important to know (especially in terms of the chemical evolution of molecules in interstellar space) the ion formation relationships with temperature. For this reason, the study of thermal ionization seems to be very interesting. Since the main DEA channel in the case of the molecules under consideration is associated with the formation of a negative oxygen ion, we also focused on identifying this pathway in our studies.

## 2. Results

**Figure 1:**
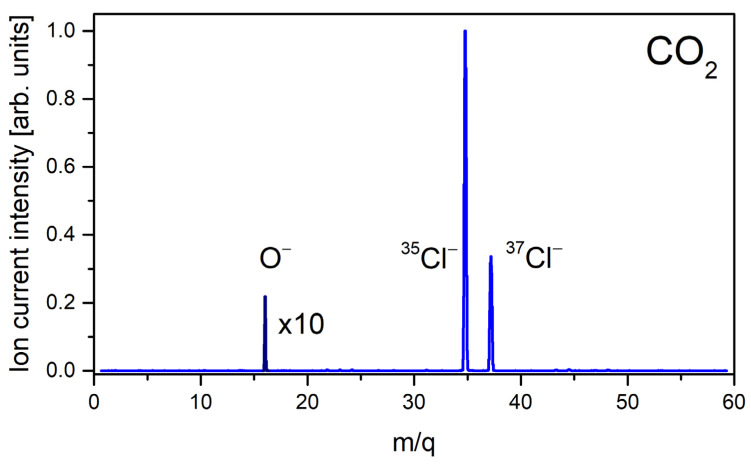
The negative ions mass spectrum obtained for mixture of CO_2_ and CH_3_Cl. The O^−^ signal is multiplied by factor 10.

In the investigations of DEA involving O_2_, CO_2_, CO, NO_2_, and NO gases, the only anion detected in the resulting mass spectrum was O^−^. This anion was formed with relatively low intensity but allowing for further studies on its formation as a function of filament/gas temperature. In fact, in our mass spectrum, the Cl^−^ anions (for ^35^Cl and ^37^Cl isotopes) which originate from CH_3_Cl were also observed, serving as the m/z scale determination. An example of the obtained negative ion mass spectrum for the thermal ionization of CO_2_ is presented in Figure 1.

The surface ionization process that leads to the formation of negative ions is governed by the Saha–Langmuir equation, which describes the degree of ionization *α* of molecules. This equation relates the ionization fraction to various parameters, including temperature and the energy properties of the particles and ionizing surface involved. The Saha–Langmuir equation can be expressed as follows:

(1) $$\alpha =\frac{N^{-}}{N_{0}}=\frac{g_{-}}{g_{0}}exp\left[\frac{EA-\varphi }{kT}\right]$$

where *N^−^* and *N*_0_ correspond to the numbers of the negatively ionized and neutral molecules, respectively, *g_−_/g*_0_ is the ratio of statistical weights of the anion and neutral molecule, *EA* is the electron affinity of the molecule, *φ* is the work function of a surface, and *k* and *T* are the Boltzmann constant and the temperature, respectively.

This equation illustrates how the degree of ionization changes with temperature and the difference between the EA and *φ*, providing insight into the conditions necessary for effective negative ion formation during surface ionization processes. Following the Saha–Langmuir equation (Equation (1)), it can be concluded that for efficient anion formation, the filament material should have a work function *φ* that is either lower than or comparable to the electron affinity (EA) of the studied molecule. More specifically, the probability of anion formation depends exponentially on the difference between the electron affinity of the molecule and the work function of the surface.

For all of the studied gas molecules, the electron affinity is relatively low, with a value ranging between of −0.60 eV and 2.27 eV (see Table 1), which is definitely smaller than the work function of the MoRe filament used (4.23 eV). To improve the efficiency of anion generation from the investigated gas samples, the ionization chamber is made of tantalum, which has a work function of 4.25 eV [31]. Since the ionization chamber is positioned very close to the filament, it can be heated to elevated temperatures both through radiation from the filament and by heat conduction. Consequently, the ionization chamber can also act as an additional ionization surface. However, despite this modification, the relatively large difference between the work function of the active surfaces in the ionization chamber and the electron affinity of studied gases results in a relatively low efficiency of anion formation.

**Figure 2:**
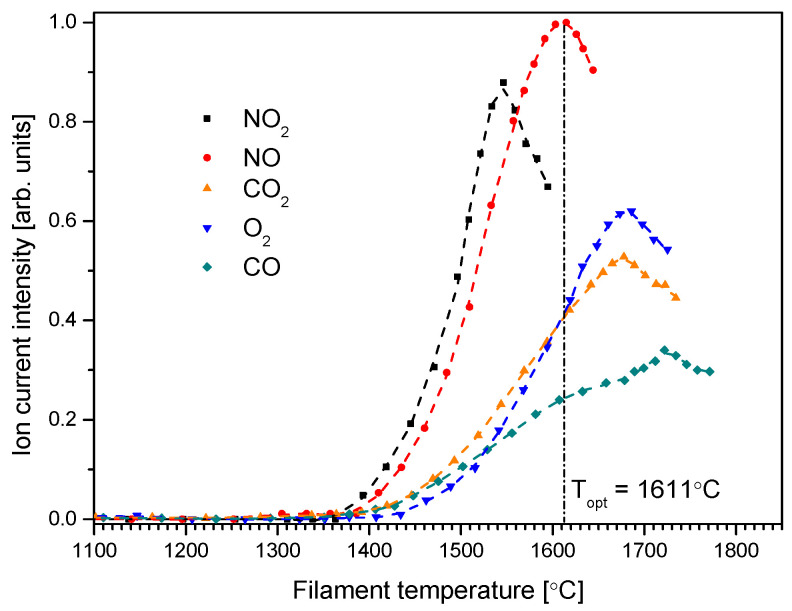
The measured O^−^ ion current intensity in function of the filament temperature.

The results of the study of O^−^ formation from the compounds considered are shown in Figure 2. This graph shows the dependence of the O^−^ ionic current intensities on the temperature of the spiral cathode (filament) for anions formed from considered gases. The obtained dependence is characterized by the occurrence of a maximum for a certain temperature. Similar dependencies of negative ion formation from the gases were also observed previously [31,34,35,36]. This temperature is the optimal temperature for the generation of the O^−^ anion (as an example, marked by vertical dash-dot line for O^−^/NO); this means that at this temperature, the formation of O^−^ is most efficient. It can be noted that although the curves relate to the intensity of O^−^ ionic currents of the same atom (but from different gases), they have both different values of ionic current intensities (at the same gas pressure in the inlet system) and different optimal temperatures for the formation of the O^−^ anion. Although the gas pressure in the dosing system was the same, the measured O^−^ ion current intensities do not directly relate to the cross section for the DEA process for the investigated molecules. Supplying different gases to the cathode area also causes modification (e.g., by the oxidation) of the cathode surface, changing the work function and, automatically, the degree of ionization (see Equation (1)).

Formation of O^−^ from the gases considered may be described by the following DEA reaction channels:

O_2_ + e^−^ → O^−^ + O   (Eth = 3.71 eV), (2)

CO_2_ + e^−^ → O^−^ + CO   (Eth = 4.06 eV), (3a)

CO_2_ + e^−^ → O^−^ + C + O   (Eth = 16.65 eV), (3b)

CO + e^−^ → O^−^ + C   (Eth = 9.63 eV), (4)

NO_2_ + e^−^ → O^−^ + NO   (Eth = 1.72 eV), (5a)

NO_2_ + e → O^−^ + N + O   (Eth = 8.26 eV), (5b)

NO + e^−^ → O^−^ + N   (Eth = 4.99 eV) (6)

For triatomic molecules, two dissociation processes are considered: one for single bond breaking ((3a) and (5a)) and second for total molecule fragmentation ((3b) and (5b)). Additionally, for every DEA path, the energy thresholds are provided here in parentheses (Eth) and were calculated on the basis of the data collected in Table 1. Obviously, the breaking of more bonds in a molecule (reactions (3b) and (5b)) are even more endothermic than reactions for the same molecules in which only one bond is broken (channel (3a) and (5a)). In the case of NO_2_ and CO_2_, more than three times as much energy is required for the complete fragmentation of the molecule as for the dissociation of these molecules into two fragments (see Table 1). In Table 1, other data regarding considered molecules and atoms are also presented.

## 3. Discussion

The O^−^ anion is a common anion observed in DEA studies on several types of oxygen containing molecules, from very complex [11,37,38] to very simple [13,14,15,39,40] ones. In the previous studies of negative ion formation from the O_2_, CO_2_, CO, NO_2_, and NO gases, the O^−^ anion was the main ion measured [12,13,14,15,16,17,18,19,20,21,22,23,24,25,26,27,28].

In the case of the generation of the O^−^ anion from the O_2_ molecule, earlier studies indicate that this is the only anion formed from this molecule for low O_2_ pressures. Previous results show that the appearance energy of O^−^/O_2_ is about 4.6 eV, while the resonance maximum occurs at an energy of about 6.6 eV [13,14]. For electron energies exceeding 17 eV, O^−^ formation is also possible as a result of the ion-pair formation process [17]. Several studies have also observed the formation of the molecular anion O_2_^−^, which could be formed by donating part of the energy by the excited (O_2_^−^)^#^ molecule, either as a radiation quantum [14] or in collisions with another molecules. T.D. Märk et al. noted, however, that O_2_^−^ can also be formed from larger (O_2_)_n_ clusters [13]. Such a process needs elevated pressures (>10^5^ Pa) and low temperatures (T < −100 °C). It should be pointed out here that in our experiment, the pressures both in the injection system (of 1000 Pa) and in the ion source chamber (*p* < 10^−3^ Pa) were significantly lower and the temperature was higher than those required for the production of (O_2_)_n_ clusters, explaining why we did not observe an O_2_^−^ ion in our study.

The DEA process leading to the formation of an O^−^ ion from a CO molecule is characterized by a rapid vertical onset at electron energy equal to 9.63 eV, which is equal to the thermochemical threshold for the electron capture reaction (4) [40]. In these studies, a small peak in the O^−^ curve at an energy of approximately 11 eV, was also recognized. This resonance was attributed to the formation of the O^−^ anion through the generation of neutral in an excited state. The onset of this peak aligns very well with the expected value of 10.89 eV, derived from the excitation energy of C*(1D) at 1.263 eV [24]. Moreover, Rapp and Briglia suggested that ion-pair formation leading to O^−^ anion formation is also possible for CO gas, but at energies above 19 eV [17].

For CO_2_, electron attachment leads to the formation of transient negative ions (CO_2_^−^)^#^, which can undergo dissociation to form O^−^ and CO [24]. Formation of the O^−^ ion during DEA to CO_2_ occurs for electron energies around 4.4 eV. For this process, a specious resonance peak with characteristic structures (additional peaks) is observed. The structures in the resonance peak are a direct result of the dissociation process of the temporary anion of CO_2_^−#^, which undergoes fragmentation into O^−^ and CO. The CO molecule during this process can find itself in various oscillation states (υ = 0, 1, 2,…). These changes in the oscillation excitations of the neutral fragment will cause the formation of several resonances with small differences in energy. For the example, Cicman et al. observed four resonance peaks, which can be distinguished in the 4.4 eV resonance structure. It should be added that the resonance has a range from about 3.9 eV (energy of O^−^/CO_2_ appearance) to about 5.5 eV [24].

It is also worth pointing out that in the aforementioned work, it was observed that the O^−^/CO_2_ electron attachment energy also depends on the temperature of gaseous CO_2_ and varies in a range from 3.904 eV at 245 K to 3.864 eV at 300 K. This phenomenon is due to the fact that at higher temperatures, there is a higher probability of oscillation excitation of molecules, so the energy of the DEA process will be lower.

Rapp and Briglia also observed O^−^/CO_2_ formation at higher energies, that is, with a resonance energy of 8.1 eV and an appearance energy of about 6.8 eV [17] (such resonance was also noted by Cicman et al. [24]), as well as ion pair formation for energies exceeding 22 eV. In their work, Wang et al. [25] mention two more DEA channels to CO_2_. They observed the formation of C^−^ and O_2_^−^ anion, with the thermochemical energy thresholds for these processes being 15.28 eV and 11.0 eV, respectively. The cross-sections for the formation of these ions are also decidedly smaller (10^4^ times) than for the DEA O^−^/CO_2_ process. For this reason, and the requirements of high TIMS temperatures for such high energies, these ions were not observed in our study.

A very interesting study of the formation of the O^−^ anion from CO_2_ adsorbed on various surfaces was conducted by Huels et al. [41]. They observed that the DEA process is highly dependent on the specific environment of the initial transient molecular anion. With the change in the composition of the substrate, the yield of negative anions from specific co-adsorbents can be slightly reduced, completely inhibited, or strongly increased. Additionally, interactions of DEA major anion fragments after their dissociation with substrate molecules can also lead to the formation of new or hybrid anion species. The resonance energy also depends on the type of surface on which the DEA process proceeds.

Nitric oxide (NO) and nitrogen dioxide (NO_2_) exhibit unique electron interaction properties due to their unpaired electron and open-shell electronic structures. Moreover, the NO molecule is similar to O_2_ one, in that the extra electron occupies an antibonding molecular orbital.

The results of Rapp and Briglia’s research show that when an electron is attached to NO, negative ions are formed (without indicating of their type) in a rather wide range of resonance energies—between about 6.6 eV and 11 eV [17]. Additionally, they observed two maxima in the resonance curve at about 8.0 and 8.7 eV. Among the studies on the formation of negative ions from NO, we should also mention the research of Hiraoka et al. [29]. In their paper, they showed that the formation of not only O^−^ but also a long-lived N^−^ ion is possible in a DEA process to NO. The O^−^ anion is formed in resonance with onset at 7.39 ± 0.05 eV and the maximum at 8 eV. In turn, the N^−^ ion has an appearance energy of 1 eV higher than that specified for O^−^ and is about 8.4 eV. In this work, the energy threshold for the formation of O^−^ ion in the process of ion pair formation was also determined as 19.9 eV. Theoretical calculations also indicate the possibility of long-lived N^−^ anion formation [20]. More recent studies do not confirm the generation of N^−^ anions from NO_2_ [12,30]. In these investigations, the onset of O^−^ formation was found for a resonance energy of 7.45 eV. The resonance peak also has broad maximum between 7.5 and 9.5 eV, indicating that O^−^ can be formed in two DEA processes with a neutral fragment (N atom) in different excitation states. We should also emphasize that in our experiments, the only observed ion formed from NO was O^−^; we did not detect the formation of N^−^ ion. It can also be pointed out here that the nitrogen anion was also not observed in studies of electron attachment to (NO)_n_ clusters. Instead, a signal from the NO^−^ monomer was measured in these studies [16].

In the context of the formation of negative ions from NO, the total cross section (TCS) for electron scattering on the NO molecule presented in the work of Song et al. is intriguing [42]. The recommended TCS presented by these authors is characterized by two strong maxima (for low electron energies), at about 1.2 eV and 10.8 eV [42]. These energies do not correspond to those observed in the DEA process, showing that at these energies proceed stronger electron scattering that does not lead to the negative ion formation.

NO_2_, with its asymmetric electronic distribution, has a higher electron affinity compared to NO, making electron attachment studies particularly important. Negative ions formed from NO_2_, such as NO_2_^−^, are relevant in atmospheric processes. Moreover, understanding electron-induced dissociation of NO_2_ provides insights into the formation of NO and O^−^ in atmospheric reactions, impacting both air quality and climate. The TCS of electron scattering for the NO_2_ molecule shows that there are two main energy regions where the DEA process may be active. They are below 1.2 eV and between 7 eV and 13 eV [42,43]. Rallis and Goodings, in paper from 1970, observed two peaks indicating DEA to NO_2_, with onset values of 1.6 ± 0.2 eV and 7.3 ± 0.3 eV, and peak values of 3.0 ± 0.2 eV and 8.1 ± 0.2 eV, respectively [23]. More recent studies exhibit that the O^−^ may be formed from NO_2_ at three major resonances with maxima at about 1.4 eV, 3.1 eV, and 8.5 eV. Moreover, an additional low resonance peak at the tail of the 8.5 eV resonance was observed with a maximum around 11 eV [22]. These findings are in line with the earlier results of Rangwala et al. for the main peaks with a small change for the resonance at 8.5 eV, which, in their work, was estimated to be 8.3 eV [26]. Discussing the formation of negative ions from NO_2_, one can also mention high-pressure experiments in which the excess energy brought into the system by an extra electron was transferred in a collision to another molecule. In such conditions, the formation of the negative parent ion NO_2_^−^ was also observed [44]. In our study at low-pressure conditions, the only anion measured from NO_2_ was O^−^.

It should be emphasized here that despite employing a completely different ionization method (thermal ionization), we observed the same ionization channels as those reported in the most recent experiments using crossed molecular and electron beams. In both our study and the latest research utilizing alternative ionization techniques, the only anion observed from the gases under investigation was the oxygen anion. Similar conclusions can also be drawn from our other studies on the thermal ionization of molecules [35,36].

To explain the occurrence of maxima in the temperature dependence of the ionic current (see Figure 2) for the formation of the O^−^ anion from the compounds under consideration, we should take into account a few processes. Firstly, thermal ionization on the surface of a heated metal (in our experiments the cathode and the ionization chamber), governed by the Saha–Langmuir law (Equation (1)); secondly, thermal dissociation of the molecule (e.g., on a heated cathode surface) and the subsequent attachment of an electron to the fragments created in this process; and thirdly, the capture of a free electron (emitted by thermo-emission) by the molecule. All these processes may lead to the formation of negative ions in our experiment.

The thermal dissociation equilibrium constant Kp (T) for dissociation reactions (2 to 6) at temperature T can be calculated using the following equation:

(7) $$K_{p}\left(T\right)={\left(\sqrt{\frac{2\pi }{h^{2}}}\right)}^{3}{\left(kT\right)}^{\frac{5}{2}}{\left(\frac{m_{P}}{m_{S}}\right)}^{\frac{3}{2}}\frac{Z_{vibP}Z_{rotP}}{Z_{vibS}Z_{rotS}}\frac{g_{P}}{g_{S}}e^{-\frac{E_{D}}{kT}}$$

where h is Planck’s constant; m_P_ and m_S_ are the masses (or multiplication of masses) of the dissociation products (P) and substrates (S), respectively; k is the Boltzmann constant; T is the temperature; and Z_vibP_, Z_rotP_, Z_vibS_, Z_rotS_, and g_P_ and g_S_ represent the vibrational and rotational partition functions, as well as the statistical weights of the ground states (or multiplication) of the reaction products (P) and substrates (S), respectively. E_D_ is the respective energy needed for fragmentation of the molecule in agreement with the respective reaction channel (2)–(6)—dissociation energy. The partition functions, and thus K_p_ (T), were calculated using the data collected in Table 1.

The degree of molecular dissociation (D_d_) was derived from the calculated equilibrium constant K_p_ (T) and the total gas pressure (p), based on the formulas of the definitions of Kp(T) and D_d_:

(8) $$K_{p}\left(T\right)=\frac{p_{1}*p_{2}}{p_{S}}$$

(9) $$D_{d}=\frac{p_{1}+p_{2}}{p_{S}+p_{1}+p_{2}}$$

where the partial pressures of the respective dissociation reaction products and considered substrate gas are denoted as p_1_, p_2_, and p_S_, respectively. Taking into account Equation (9), one can see that the D_d_ is equal to zero when no molecule dissociation occurs, and conversely, D_d_ = 1 when all of the substrate molecules are fragmented. In the case of CO_2_ and NO_2_, the dissociation energies for the complete fragmentation of molecules during a single-step process (the reactions described by Equations (3b) and (5b)) are far larger than for the energies needed to break a single bond in a molecule. Considering this fact, it is safe to say that the occurrence of a one-step total dissociation process will have a much lower probability than processes in which the dissociation of CO_2_ and NO_2_ molecules will take place in steps through breaking successive bonds with oxygen atoms in the molecule. For this reason, in our subsequent considerations, we will omit the processes of total one-step dissociation ((3b) and (5b)) for both molecules mentioned above.

**Figure 3:**
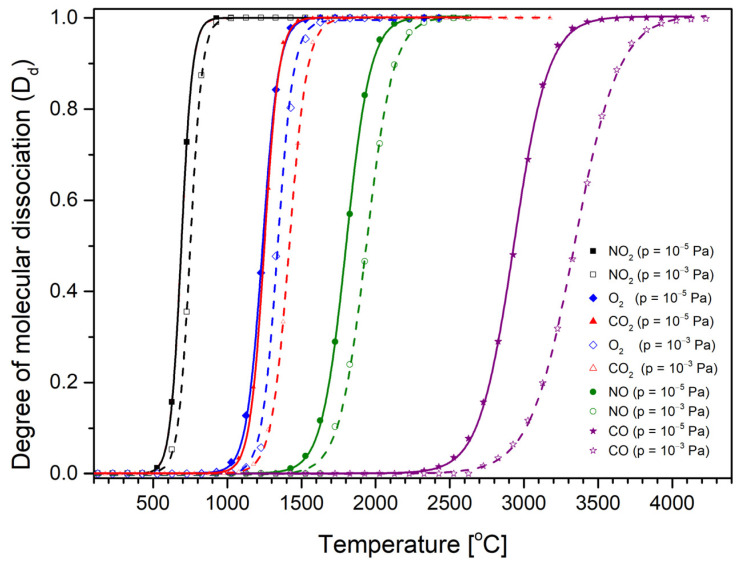
The calculated dissociation degree of O_2_, CO_2_, CO, NO, and NO_2_ molecules in the gas phase as a function of the gas temperature. Two pressures of the gas were taken into consideration: *p* = 10^−3^ and 10^−5^ Pa.

The usual operating pressure in the ion flight tube during measurement is around 10^−^⁵ Pa. However, the gas pressure in the ionization chamber must be considerably higher, as the gas is directly introduced into this chamber. For this reason, we considered two gas pressures, 10^−^⁵ Pa and 10^−3^ Pa, in the D_d_ calculations. We selected two extreme pressure values, as the pressure in the ionization chamber is expected to be within the range between them. Calculated on the basis of Equations (7)–(9), the dissociation degrees of single oxygen atoms from the O_2_, CO_2_, CO, NO, and NO_2_ molecules as a function of the gas temperature at two pressures of 10^−5^ Pa and 10^−3^ Pa are shown in Figure 3.

The calculated degree of dissociation increases with temperature. From Figure 3, we can conclude that each gas undergoes thermal dissociation at a different temperature. For example, (for a pressure of 10^−3^ Pa) NO_2_ begins to dissociate (D_d_ = 0.01) at a temperature of about 500 °C, and at a temperature of about 900 °C, it is already in the fully dissociated form (NO + O). Similar relationships exist for the other gases under consideration, i.e., for O_2_ and CO_2_, the beginning of dissociation takes place at around 1070 and 1080 °C, respectively. The highest temperatures required for the beginning of fragmentation are necessary for CO (2570 °C). These data are collected in Table 2. In Table 2, we also provide the values of the measured optimal temperatures for O^−^ formation and the single oxygen atom dissociation energy and obtained in the earlier experimental studies of O^−^ appearance energies from different gases. In addition, we can observe that for lower gas pressures, the temperature of its decomposition is also lower. In general, we can say that the calculated temperature of the beginning thermal fragmentation (D_d_ = 0.01) of a molecule is lower for gases with lower dissociation energy (see Table 2). This assumption is in line with the earlier observation in studies of anion formation from the SF_6_ molecule. In that paper, it was stated that the optimal temperature depends on the threshold energies of the respective anion formation thus is connected to both the dissociation energy and the electron affinity of the generated fragments [35].

In our study, we measure the intensities of the same type of ion (O^−^) currents. For this reason, the optimal temperature of its formation should depend only on the dissociation energy of the molecule or, alternatively, on the energy of the appearance of O^−^ for its generation from different gases.

**Figure 4:**
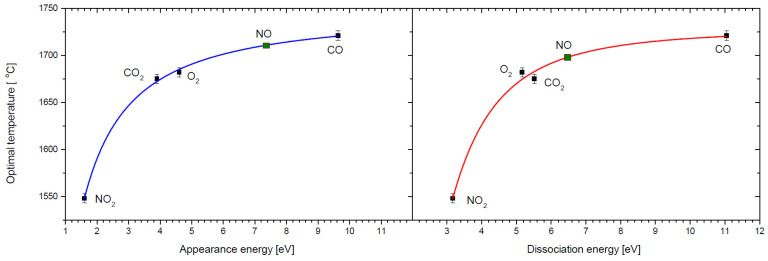
The dependence of optimal temperature for O^−^ formation for CO_2_, CO, O_2_, and NO_2_ on the experimental appearance energy of O^−^ (blue line, left side) and on the oxygen atom dissociation (red line, right side) for the considered molecules. Green points correspond to the position of NO molecule on the respective plot.

There is one deviation from this rule in the current study, namely, in the case of NO molecules with a dissociation energy of 6.54 eV, the measured optimal O^−^ formation temperature (equal of 1611 °C) is significantly lower (at least 60 °C) than in the case of O_2_ or CO_2_, who possess lower dissociation energies of 5.17 and 5.52 eV, respectively. We must mention, however, that in the case of this gas (NO), there were great difficulties in dispensing it, probably due to air contamination of the gas in the cylinder we had. The gas collected in the glass vessel was slightly brown in color—which is a characteristic color for NO_2_ gas. NO reacts very easily with oxygen, resulting in the formation of NO_2_ [45]. Therefore, in our experiments, we had a mixture of NO and NO_2_ with an unknown ratio. This means that real optimal temperature for O^−^ formation from NO should be significantly shifted to higher values, as NO_2_ dissociation energy is lower than that for NO. We tried to estimate the real optimal temperature for clean NO based on the data on O^−^ formation for other molecules. Figure 4 presents the obtained dependencies of optimal temperature on the O^−^ experimental appearance energies and oxygen bond dissociation energy for the studied molecules (see Table 2).

Obtained on the basis of these plots, the optimal temperatures for oxygen anion formation from NO are 1711 °C and 1698 °C, respectively, giving an average value of 1705 °C. Another interesting observation arising from the comparison of experimental (optimal temperature) and theoretical (degree of dissociation) results is that, at first sight, we do not observe a close relationship between both temperatures: optimal and dissociation. If the process of negative surface ionization were only associated with the dissociation of molecules, then the optimal temperature should be greater than the temperature at which all molecules have dissociated. This is the case for NO_2_, O_2_, and CO_2_. For the CO molecule, however, the situation is the opposite—the optimal temperature is several hundred degrees Celsius lower than the temperature of complete dissociation of this molecule. In addition, in the case of CO_2_ and O_2_, calculations indicate that D_d_ = 0.99 at a temperature of about 1650 °C. This temperature basically coincides with the optimal temperature for the formation of O^−^ from these gases. For NO_2_, the situation is different—the optimal temperature is significantly higher (by about 500 °C) than the total dissociation temperature of this molecule (~980 °C). This suggests that perhaps there is another process associated that leads to the thermal decomposition of this molecule. The previously suggested channel for the thermal dissociation of NO_2_ is as follows [46]:

NO_2_ → O_2_ + N. (10)

This means that in the reaction (10), the O_2_ molecule is generated, which then may undergo further fragmentation to form an O^−^ anion. The dissociation energy requested for reaction (10) is equal to 0.59 eV and is much lower than for other channels (3–6). If we assume that the formation of O^−^ from a NO_2_ molecule is a two-step process, i.e., firstly reaction (10) occurs, followed by reaction (2), then the total energy required for both reactions is 4.3 eV. Considering this energy, the optimum temperature for O^−^ formation from NO_2_ should be higher than the temperatures required for the formation of oxygen anions from CO_2_ and O_2_, which, according to reactions (2) and (3a), require 3.71 eV and 4.06 eV, respectively.

To explain the lower optimum temperature observed for NO_2_ (despite the two-step dissociation process), it should be noted that during the dissociation described by Equation (10), the O_2_ molecule can be generated already in an excited state. As a result, it requires less energy to dissociate than that indicated by reaction (2); therefore, its optimal O^−^ formation temperature may have a lower value than in the case of CO_2_ and O_2_.

In the case of O^−^ formation from CO, on the other hand, the optimum temperature (1721 °C) is much lower than even the temperature at which this molecule begins to dissociate (D_d_ = 0.01 for 2280 °C). This means that the process of O^−^ formation from CO is not directly related to the thermal decomposition of the CO molecule. In this regard, we must also take into account the direct attachment of an electron to a CO molecule and the subsequent dissociation process leading to the formation of O^−^ (dissociative electron attachment—DEA). Such an option of anion formation was also proposed earlier in the case of studies of SF_6_ [35]. In such a process, the energy of the captured electron must match the resonance energy of DEA [11]. For CO, this energy is 9.63 eV. Electrons emitted in the process of thermo-emission from the cathode can be accelerated to such energies in an electric field. To achieve a filament temperature of 1721 °C, a voltage of 7.8 V is applied to the ends of the cathode. However, this difference in potentials is too low for the electron to obtain the resonance energy for the DEA process (O^−^/CO). We must remember, however, that the ion source of the applied mass spectrometer operates in the negative ion beam forming mode, i.e., potentials are applied to form and accelerate the negative ion beam. These potentials can also cause electrons to obtain resonance energies required for the DEA process. Thus, the formation of O^−^ from CO may be possible even at temperatures below the thermal decomposition of the CO molecule.

## 4. Experiments

The experimental setup has been previously described in detail elsewhere [31,47]; a brief overview is provided here. The mass spectrometer employed in this study is composed of four primary components: a gas inlet system, an ion source, a magnetic sector, and a detection system. Specifically, a negative thermal ionization mass spectrometry (TIMS) ion source is applied.

Gas pressure in the inlet system is monitored using a Pirani gauge, while the pressure in the main chamber of the mass spectrometer is measured by an ionization gauge. Pure studied gas (one of O_2_, CO_2_, CO, NO_2_, NO) is introduced into the ionization chamber from the gas inlet system via two segments; the first is a 1 m long stainless steel capillary tube with an internal orifice diameter of 70 μm, and the second is a 0.10 m long quartz tube with an internal diameter of 2 mm. The quartz part also provides electrical isolation of the ion source from the rest of the equipment located at ground potential. Prior to the introduction of gas under consideration, the entire inlet system and capillary is heated for several hours at about 80 °C.

The ionization chamber itself is cylindrical and constructed from tantalum. Inside the chamber, a spiral filament, made of the MoRe alloy wire (Mo52.5/Re47.5), is installed. Previous studies have shown that the use of a MoRe alloy cathode is the most optimal in terms of the stability and duration of operation (without burning out of the cathode) of the ion source [31]. One end of the filament is electrically connected to the ionization chamber, while the other end is connected to an extraction electrode, located at the exit of the ionization chamber. The extraction electrode, made of tantalum, features a narrow slit whose height corresponds to the internal diameter of the ionization chamber.

In this configuration, the studied gas flows around the heated filament. Filament temperature, with an uncertainty of ±5 °C, is measured via pyrometry. In order to correct the pyrometer readings, the emissivity of the cathode material (MoRe) was set to 0.34 as an average of the emissivity values for pure materials: molybdenum and rhenium [48]. The pyrometer is mounted outside the vacuum chamber of the mass spectrometer. Thermal radiation reaches the pyrometer through a vacuum quartz viewport. The focal point of the pyrometer’s collecting lens is aligned with the surface of heated cathode.

**Figure 5:**
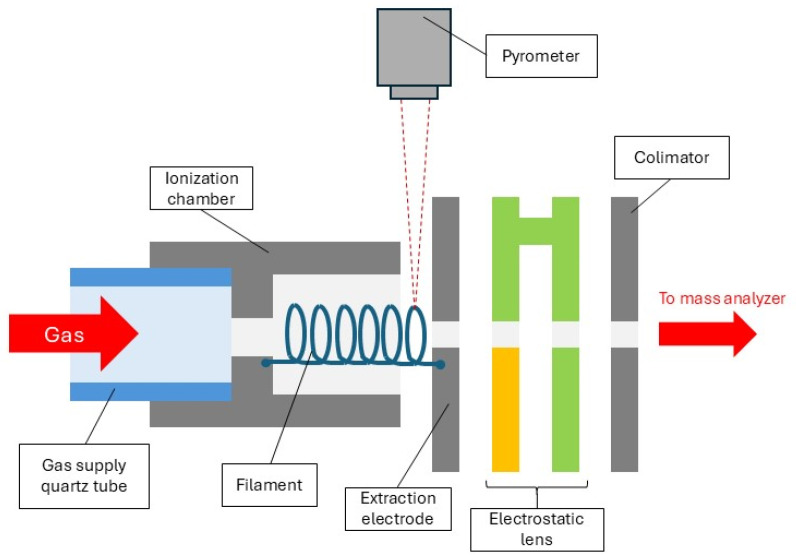
A schematic diagram of the ion source employed in the present study.

Molecules near the filament may undergo dissociation or/and electron capture, leading to the formation of negative ions. A high voltage of −3 kV is applied to the extraction electrode, accelerating the generated anions. Figure 5 shows a schematic view of the ion source used in the experiments described.

The accelerated ions are directed towards an electrostatic lens, which is maintained at a potential slightly higher (~300 V) than the extraction electrode. After passing through the electrostatic lens, the ion beam is focused onto a collimator slit at ground potential. The beam is subsequently analyzed within the magnetic sector and detected by the detector system. To improve anion detection efficiency, a channeltron detector (Dr. Sjuts, Optotechnik GmbH, Goettingen, Germany) coupled with an appropriate counting system is employed. The mass spectrometer used in the study was built from scratch in our laboratory. The O_2_, CO_2_, CO, NO_2_, NO gases, with a purity of 99.98%, were sourced from Merck, Darmstadt, Germany.

## 5. Conclusions

It should be noted that surface ionization is a relatively simple and effective method of negative ion generation, especially when compared with crossed-beam experiments, in which the monochromatization of electron energy is required. It is also noteworthy that the results obtained (types of ions generated) coincide with those obtained in crossed-beam experiments on molecules and electrons. Here, we do not obtain the exact energy data related to the resonance energies for the DEA process, but by considering the optimal temperatures, we can estimate the relationship between the dissociation energies of the molecules.

Given their significance in environmental, atmospheric, and biological contexts, a detailed examination of O_2_, CO_2_, CO, NO, and NO_2_, including their electron interactions and attachment properties, is crucial. In our research, we addressed the formation of negative oxygen ions from several simple gases. The dependence of the intensity of the O^−^ ion current as a function of the filament temperature was studied. For all gases, optimum O^−^ ion formation temperatures were determined, which, depending on the gas, range from 1548 to 1721 °C. Comparison of the optimal temperatures of O^−^ generation with the calculated temperatures of thermal decomposition of the studied compounds showed that the different processes of formation of this ion are involved for each of the studied gases. In the case of NO_2_, the process can proceed through two steps of dissociation, where in the first step O_2_ is formed, dissociating in the second step to O^−^ and O. For the formation of O^−^ from CO, the dominant process is electron capture into the molecule further leading to dissociation, in which the O^−^ ion is generated. Hence, it should be noted that the process of so-called negative surface ionization of gases is complex and is also connected with the capture of a free electron emitted from a heated cathode. For better understanding of negative ion formation, other studies on negative thermal ionization should be conducted.

## Figures and Tables

**Table 1:** Physical and chemical properties of molecules and atoms entangled in oxygen anion formation in the described studies.

Molecule/Atom	Electron Affinity [eV] [32]	Δ_f_H°_gas_ [kJ/mol] [32]	Vibrational Frequencies [cm^−1^] [33]	Moment of Inertia [kgm^2^] [33]	Dissociation Energies [eV]
O_2_	0.45		1556.4	1.946 × 10^−46^	5.17
CO_2_	−0.60	−393.52	2348 1333 667	7.576 × 10^−46^	5.52 (CO + O) 18.11 (C + 2O)
CO	1.33	−110.53	1050	1.47 × 10^−46^	11.05
NO_2_	2.27	33.10	1318 760 1618	6.76 × 10^−46^ 6.76 × 10^−46^ 3.42 × 10^−47^	3.17 (NO + O) 9.72 (N + 2O)
NO	0.026 [12]	90.29	1876	1.64 × 10^−46^	6.54
O	1.46	249.18			
C	1.26	716.67			
N	−0.07 [34]	472.68			

**Table 2:** The obtained data regarding studied molecules: the optimal and dissociation beginning (D_d_ = 0.01) temperatures, dissociation energies for single oxygen atom bond disruption, and the experimental appearance energies for O^−^ formation from the molecules under consideration.

Molecule	Optimal Temperature for O^−^ Formation [°C]	Calculated Temperature [°C] of Dissociation for D_d_ = 0.01	Oxygen Atom Dissociation Energies [eV]	Experimental Appearance Energies [eV] (For O^−^ Formation)
NO_2_	1548 ± 5	500	3.17	1.6 [23]
CO_2_	1675± 5	1080	5.52	3.9 [24]
O_2_	1682 ± 5	1070	5.17	4.6 [13]
CO	1721 ± 5	2570	11.05	9.63 [41]
NO	1611 ± 5	1450	6.54	7.39 [30]

## Data Availability

The data presented in this study are available on request from the corresponding author.
